# Knockout of Vasohibin-1 Gene in Mice Results in Healthy Longevity with Reduced Expression of Insulin Receptor, Insulin Receptor Substrate 1, and Insulin Receptor Substrate 2 in Their White Adipose Tissue

**DOI:** 10.1155/2017/9851380

**Published:** 2017-03-06

**Authors:** Eichi Takeda, Yasuhiro Suzuki, Tetsuya Yamada, Hideki Katagiri, Yasufumi Sato

**Affiliations:** ^1^Department of Vascular Biology, Institute of Development, Aging, and Cancer, Tohoku University, Sendai, Japan; ^2^Department of Diabetes and Metabolism, Graduate School of Medicine, Tokyo University, Tokyo, Japan

## Abstract

Vasohibin-1 (Vash1), originally isolated as an endothelium-derived angiogenesis inhibitor, has a characteristic of promoting stress tolerance in endothelial cells (ECs). We therefore speculated that the lack of the* vash1* gene would result in a short lifespan. However, to our surprise,* vash1*^−/−^ mice lived significantly longer with a milder senescence phenotype than wild-type (WT) mice. We sought the cause of this healthy longevity and found that* vash1*^−/−^ mice exhibited mild insulin resistance along with reduced expression of the insulin receptor (insr), insulin receptor substrate 1 (irs-1), and insulin receptor substrate 2 (irs-2) in their white adipose tissue (WAT) but not in their liver or skeletal muscle. The expression of vash1 dominated in the WAT among those 3 organs. Importantly,* vash1*^−/−^ mice did not develop diabetes even when fed a high-fat diet. These results indicate that the expression of vash1 was required for the normal insulin sensitivity of the WAT and that the target molecules for this activity were insr, irs1, and irs2. The lack of vash1 caused mild insulin resistance without the outbreak of overt diabetes and might contribute to healthy longevity.

## 1. Introduction

The average human lifespan has been substantially elongated due to the improvement of public health and medical care in developed countries. Aging is associated with numerous changes to systemic physiology that affect physical function and performance. Moreover, aging is closely associated with various pathological conditions such as cancer and cardiovascular and neurodegenerative diseases. For this reason, aging has now become the greatest risk factor for disability and mortality, and healthy longevity has become one of the major objectives of medical research [1].

Numerous studies have being focused on the mechanism of aging [2], and so far they have revealed certain genes and signaling pathways that contribute to the regulation of the lifespan [3, 4]. Among them, insulin signaling plays a crucial and evolutionally conserved role in longevity [5, 6]. Decreased insulin signaling for longevity was first demonstrated in* C. elegance* [7] and next* Drosophila* [8] and further confirmed in mammals [9]. Indeed, the fat-tissue specific knockout of the* insulin receptor* gene in mice results in an extended lifespan [9]. However, as insulin signaling is essential for glucose homeostasis, this decreased insulin signaling should cause diabetes mellitus. Such a paradox has raised a challenging question as to how the preserved insulin signaling for glucose homeostasis and the reduced insulin signaling for longevity can be balanced [10].

The vascular system is one of the major target organs affected by aging [11, 12]. In order to maintain vascular integrity, vascular endothelial cells (ECs) should have self-defense systems. We previously reported that vasohibin-1 (Vash1) could be one of such systems [13]. Vash1 was originally isolated as an angiogenesis inhibitor was preferentially expressed in ECs for negative-feedback regulation [14]. However, our subsequent analysis revealed that Vash1 has an additional function that causes an upsurge in stress resistance of ECs by increasing the expression of superoxide dismutase 2 (SOD2) and SIRT1 in ECs [15]. Along with this finding, we observed that the decreased expression of Vash1 promotes vascular diseases such as diabetic nephropathy and atherosclerosis [16, 17]. We then noticed that the expression of Vash1 in ECs is downregulated with aging due to an increase in the expression of a certain microRNA, namely, miR-22 [17]. This observation raised the question as to why nature would allow a decrease in the expression of such a valuable protein with aging.

Because of the protective role of Vash1 in the vasculature, in this present study we assumed that* vash1*^−/−^ mice would have a short lifespan. However, to our surprise,* vash1*^−/−^ mice lived significantly longer and looked healthier than wild-type (WT) mice. Therefore, we examined the reason for this unexpected healthy longevity in* vash1*^−/−^ mice in relation to insulin signaling and further considered the meaning of this age-associated downregulation of vash1.

## 2. Materials and Methods

### 2.1. Quantitative Reverse Transcription Real-Time Polymerase Chain Reaction (qRT-PCR)

Total RNA was prepared from mouse tissues by using ISOGEN II (Nippon Gene, Tokyo, Japan) according to the manufacturer's instructions. Single-stranded complementary DNA (cDNA) was synthesized by using ReverTra Ace (TOYOBO, Tokyo, Japan). PCR was performed with a thermal cycler system (CFX-96 Real-Time system, C1000 Thermal Cycler, Bio-Rad, Hercules, CA, USA) and SsoAdvanced Universal SYBR Green Supermix (BIO-Rad, Tokyo, Japan). *β*-actin was used as the reference gene. The primer pairs are shown in Table 2.

### 2.2. Animal Studies

All of the animal studies were approved by the Center for Laboratory Animal Research of Tohoku University. WT and* Vash1*^−/−^ mice on a C57BL/6J background [18] were maintained under specific pathogen-free conditions with normal chow (CE-2; CLEA Japan Inc., Tokyo, Japan). WT and* Vash1*^−/−^ mice were separately maintained for a certain period of time. To exclude the possible genetic events accumulated over time during separate inbreeding, we crossed* Vash1*^−/−^ mice with WT mice, generated* Vash1*^+/−^,* and then obtained Vash1*^−/−^ again. We defined mice at the age of 8–10 weeks as young and those at 24–26 months as old.

#### 2.2.1. Excision of Certain Mouse Tissues

Selected tissues such as white adipose tissue (epididymal fat pad), liver, and skeletal muscle were excised from the sacrificed mice, snap frozen, and stored at −80°C prior to examination.

#### 2.2.2. High-Fat Diet (HFD) Feeding

Young WT and* Vash1*^−/−^ mice were fed a HFD (D12079B, Research Diets, Inc., New Brunswick, NJ, USA) for 12 weeks. After the mice had been fasted for 10 hours (daytime), blood was collected via a tail vein every 2 weeks. Fasting blood glucose levels were measured by using a Glutest mint (Sanwa Kagaku Kenkyusho, Nagoya, Japan). Plasma insulin and leptin levels were measured with an Ultra-Sensitive Mouse Insulin ELISA Kit (Morinaga Institute of Biological Science, Yokohama, Japan). Plasma adiponectin levels were measured with Mouse/Rat Adiponectin ELISA Kit (Otsuka, Tokushima, Japan). The HOMA-IR index was calculated from the fasting insulin and glucose levels by using the following formula: 26 × fasting plasma insulin (*μ*U/mL) × fasting blood glucose (mg/dL)/405.

#### 2.2.3. Intraperitoneal Glucose Tolerance Test (IPGTT)

IPGTT was performed for 10 hours (daytime) on fasted animals after 12 weeks of HFD feeding. The mice were injected with glucose (2 g/kg of body weight) intraperitoneally, after which blood glucose levels were measured at 0, 15, 30, 60, 90, and 120 min after injection.

#### 2.2.4. Intraperitoneal Insulin Tolerance Test (IPITT)

IPITT was performed on ad libitum-fed mice after 12 weeks of HFD feeding. The mice were injected with human regular insulin (0.5 or 0.75 U/kg body weight, Novolin R; Novo Nordisk, Bagsvaerd, Denmark) intraperitoneally, followed by measurement of blood glucose levels at 0, 10, 20, 40, 60, and 80 min after injection.

#### 2.2.5. Biochemical Analysis of Serum Samples

Mice were sacrificed after a 16-hour fast; and blood samples were taken by cardiopuncture. The serum was prepared by centrifugation (2000*g*, 20 min, 4°C). For measuring certain parameters, samples were outsourced to ORIENTAL YEAST CO., LTD (Tokyo, Japan) for serum analysis.

#### 2.2.6. Isolation of CD31-Positive ECs from the WAT

CD31-positive ECs were isolated from the WAT (epididymal fat pad) by the use of a Magnetic Cell Sorting System (MACS, Miltenyi Biotec, Auburn, CA). Tissues of WT or* Vash1*^−/−^ mice were minced and digested with collagenase I and Dispase II (Wako). The cell suspensions were then filtered through a 70 *μ*m cell strainer (BD) and treated with ACK Lysing Buffer (GIBCO) to remove erythrocytes. CD31-positive ECs were isolated by MACS with CD31 MicroBeads (Miltenyi Biotec) according to the manufacturer's instructions.

### 2.3. Quantification of vash1 Expression in Adipocytes

3T3L1 preadipocytes were obtained from the Japanese Collection of Research Bioresources and cultured in Dulbecco's Modified Eagles Medium (DMEM)/low glucose media containing 10% fetal bovine serum (FBS; Sigma-Aldrich). Confluent 3T3L1 preadipocytes were cultured in differentiation medium consisting of DMEM, 10% FBS, 500 *μ*M 3-isobutyl-1-methylxanthine (Wako), 10 *μ*g/mL insulin (Wako), and 1 *μ*M dexamethasone (Wako) for 3 days, after which the medium was aspirated and replaced with growth medium containing 10 *μ*g/mL insulin. After 2 days, the medium was changed to growth medium for an additional 2 days to allow 3T3L1 cells to differentiate into mature adipocytes.

Total RNA was extracted from the cells by using an RNeasy plus mini-kit (QIAGEN). First-strand cDNA was synthesized from 1 *μ*g of total RNA by use of ReverTra Ace (TOYOBO). QRT-PCR was carried out as described above. Reactions were carried out in triplicate and contained either 1 *μ*L of the first-strand cDNA products or serially-diluted mouse* Vash1* cDNA standards (1 × 10 to 1 × 105 copies). PCR conditions consisted of an initial denaturation step at 95°C for 3 min, followed by 40 cycles of 10 sec at 95°C, 10 sec at 56°C, and 30 sec at 72°C. Quantification analysis was carried out with Bio-Rad CFX Manager software (Bio-Rad Laboratories).

### 2.4. Statistical Analysis

Data were expressed as the means ± SD. The statistical significance of differences between groups and *p* values were calculated by using unpaired Student's *t*-test or by one-way ANOVA followed by the Tukey post hoc test. A value of *p* < 0.05 was the criterion for significance. The significance of the difference between survival curves was analyzed by Kaplan-Meier survival analysis with log-rank testing. A value of *p* < 0.05 was the criterion for significance.

## 3. Results

### 3.1. Vash1^−/−^ Mice Live Longer with a Milder Senescence Phenotype

**Figure 1:**
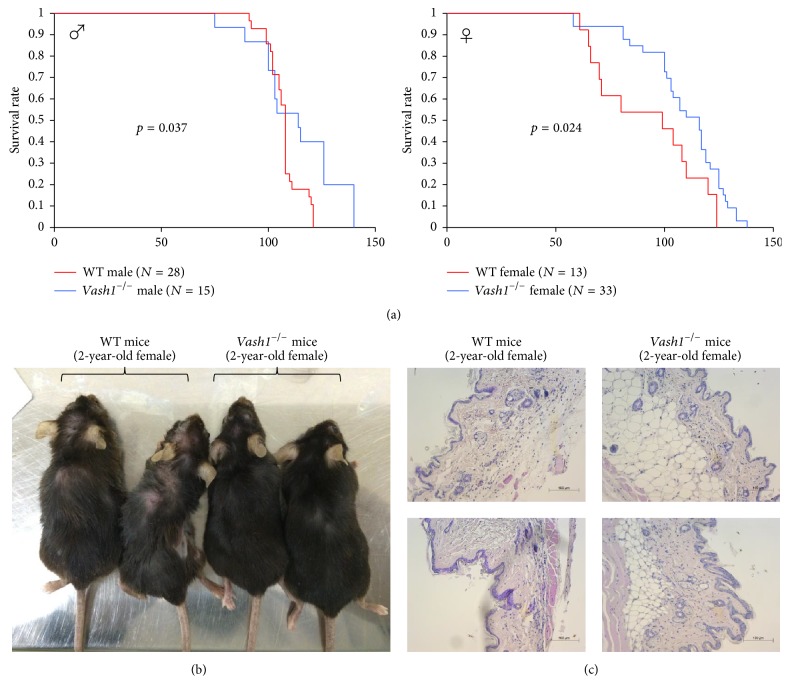
Healthy longevity of* vash1*^−/−^ mice of both genders. (a) WT and* vash1*^−/−^ mice of either gender were fed normal chow and compared for their survival rate. (b) Gross appearance of 2-year-old female WT and* vash1*^−/−^ mice is shown. (c) Subcutaneous tissues from 2-year-old female WT and* vash1*^−/−^ mice, stained with hematoxylin and eosin.

We compared the lifespan of* vash1*^−/−^ mice with that of wild-type (WT) mice. Mice were fed normal chow, and no growth retardation or abnormalities were observed in the* vash1*^−/−^ mice. We initially speculated that the* vash1*^−/−^ mice would have a short lifespan. However, to our surprise,* vash1*^−/−^ mice lived significantly longer than the WT ones. This observation was made in both male and female knockout mice, but the difference appeared earlier in the female mice (Figure 1(a)). We also noticed obvious changes in their appearance. WT female mice at 2 years of age showed a senescence phenotype with missing hair, but such phenotype was scarcely observed in the* vash1*^−/−^ female mice (Figure 1(b)). Also, the subcutaneous fat tissue became thinner in the WT female mice, whereas it was preserved in the* vash1*^−/−^ females (Figure 1(c)). Thus,* vash1*^−/−^ mice exhibited healthy longevity.

### 3.2. Mild Insulin Resistance in vash1^−/−^ Mice in Their Early Life

**Figure 2:**
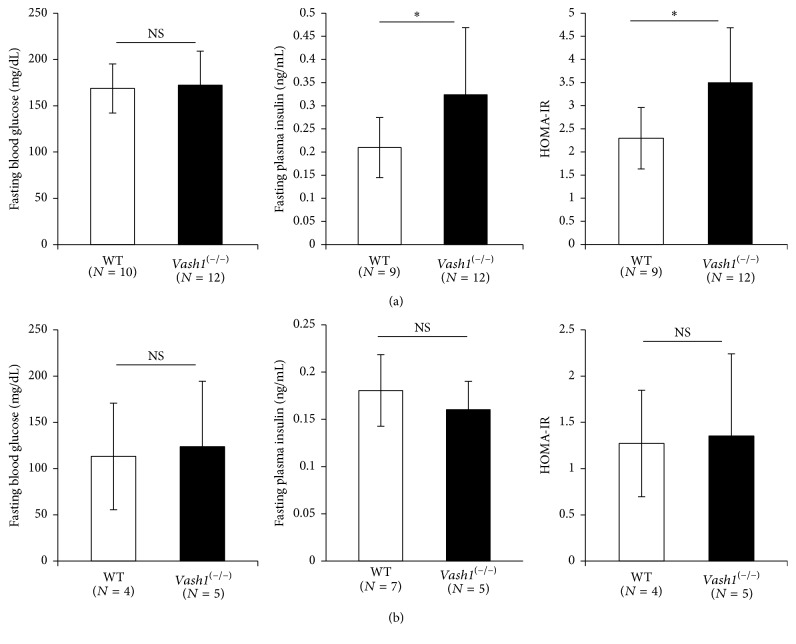
Insulin resistance in young* vash1*^−/−^ mice. Fasting blood glucose, fasting plasma insulin, and HOMA-IR are compared between WT and* vash1*^−/−^ of young (a) and old (b) male mice. Means ± SDs are shown. The statistical significance of differences was calculated by use of unpaired Student's *t*-test, and a value of *p* < 0.05 was the criterion for significance. ^*∗*^*p* < 0.05.

As insulin signaling is critically involved in the determination of lifespan; next we examined the metabolic status of the animals.* Vash1*^−/−^ mice exhibited mild but apparent insulin resistance without elevation of their blood glucose in early life (Figure 2(a)), and such changes disappeared in late life (Figure 2(b)). Histological analysis revealed no obvious changes in pancreatic islet in their early life (see Supporting Figure  1 in Supplementary Material available online at https://doi.org/10.1155/2017/9851380). Serum biochemical analysis further revealed that there were no obvious abnormal findings in the* vash1*^−/−^ mice, and besides, they had significantly lower levels of CRE, T-CHOL, F-CHOL, E-CHOL, PL, T-BIL, and D-BIL than the WT mice (Table 1).

### 3.3. Decreased Expression of insr, irs-1, and irs-2 in the WAT of vash1^−/−^ Mice

**Figure 3:**
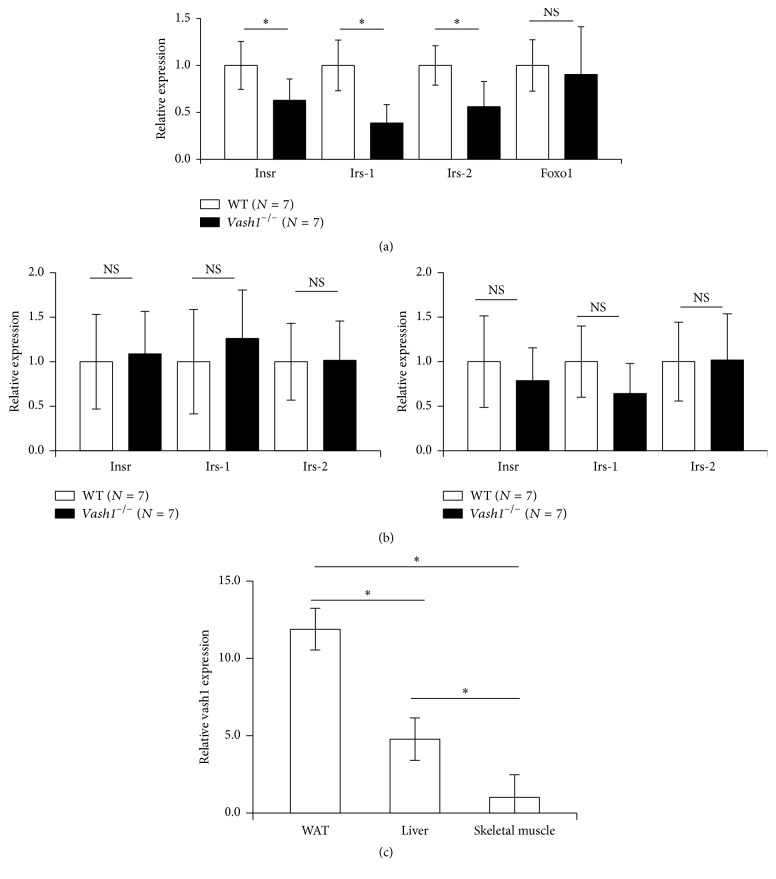
Downregulation of insr, irs-1, and irs-2 in WAT of young* vash1*^−/−^ mice. (a) Total RNA was isolated from the WAT of WT and* vash1*^−/−^ young male mice, and the expression of insr, irs-1, irs-2, and Foxo1 was compared between WT and* vash1*^−/−^ mice. (b) Total RNA was isolated from skeletal muscle or liver of WT and* vash1*^−/−^ young male mice; and the expression of insr, irs-1, and irs-2 was compared between WT and* vash1*^−/−^ mouse tissues. (c) Total RNA was isolated from WAT, liver, and skeletal muscle of young WT male mice, and the expression of vash1 was compared. In (a)–(c), the means ± SDs are shown, the statistical significance of differences was calculated by use of the unpaired Student's *t*-test, and a value of *p* < 0.05 was the criterion for significance. ^*∗*^*p* < 0.05.

Because of the presence of insulin resistance, we examined the major target organs of insulin and relevant genes that might cause insulin resistance. The expression of insulin receptor (insr), insulin receptor substrate 1 (irs-1), and insulin receptor substrate 2 (irs-2), but not that of Foxo1, was significantly downregulated in the WAT of* vash1*^−/−^ mice in their early life (Figure 3(a)). However, we could not find any significant differences in insr, irs-1, or irs-2 expression between the WT mice and* vash1*^−/−^ mice in their skeletal muscle or liver (Figure 3(b)). In order to understand the cause of these differential changes in isnr, irs-1, and irs-2 expression between WT and* vash1*^−/−^ mice among the 3 organs, we compared the level of vash1 expression in them. As shown in Figure 3(c), the expression of vash1 in the WT mice dominated in their WAT in early life. This expression pattern can be confirmed in human in the Genotype-Tissue Expression Portal (Supporting Figure  2). Thus, the distinctive expression pattern of vash1 among the 3 organs should explain such differential changes and further highlight the distinguishing role of vash1 in the WAT for the expression of insr, irs-1, and irs-2.

**Figure 4:**
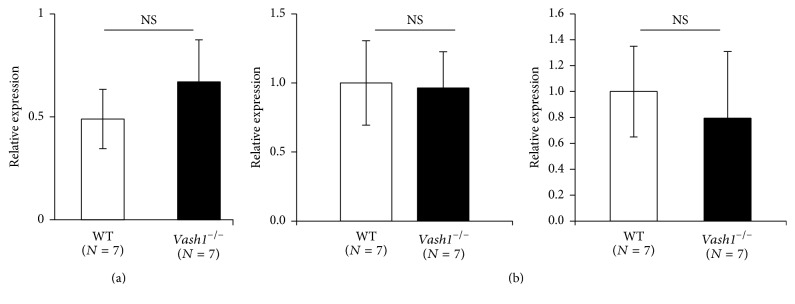
No alteration in the expression of tnf*α*, sod2, and sirt1 in the WAT of young* vash1*^−/−^ mice. (a) Total RNA was isolated from the WT and* vash1*^−/−^ WAT of young male mice, and the expression of tnf*α* was compared between WT and* vash1*^−/−^ mice. (b) Total RNA was isolated from WAT of WT and* vash1*^−/−^ of young male mice, and the expression of sod2 and sirt1 was compared between WT and* vash1*^−/−^ mice. In (a) and (b), the means ± SDs are shown, the statistical significance of differences was calculated by use of the unpaired Student's *t*-test, and a value of *p* < 0.05 was taken as the criterion for significance.

We further examined the expression of tnf*α* as an inflammatory cytokine responsible for insulin resistance, as well as sirt1 and sod2 as targets genes of Vash1 in ECs, but we could not find any differences in their expression levels between WT mice and* vash1*^−/−^ mice in their WAT (Figures 4(a) and 4(b)).

We reported previously that the expression of vash1 is downregulated with aging [17]. We confirmed this age-associated downregulation of vash1 in the WAT (Supporting Figure  3A). Therefore, the difference in vash1 expression in WAT between WT mice and* vash1*^−/−^ mice was large in their early life and minimized in their late life. Accordingly, the differences in the expression of insr, irs-1, and irs-2 in WAT disappeared in their late life (Supporting Figure  3B). We speculate this to be the reason why insulin resistance disappeared in their late life (Figure 2(b)).

**Figure 5:**
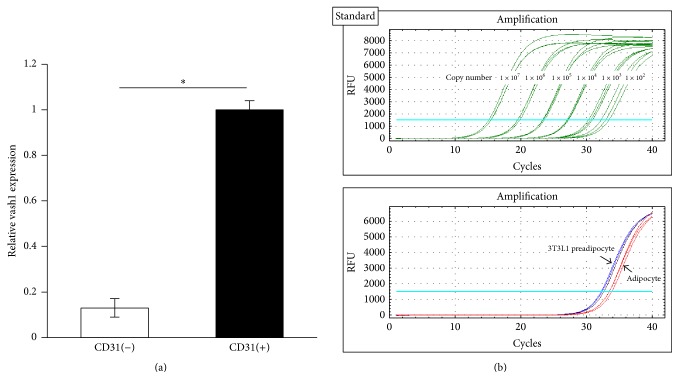
Vash1 is expressed in ECs but not in adipocytes. (a) CD31-positive and -negative cells from the WAT of young WT male mice were separated as described in Materials and Methods, and the expression of vash1 was compared. Means ± SDs are shown. The statistical significance of differences was calculated by use of the unpaired Student's *t*-test, and a value of *p* < 0.05 was the criterion for significance. (b) 3T3L1 preadipocytes were caused to differentiate into adipocyte in vitro, and the expression of vash1 in those cells was quantified as described in Materials and Methods. ^*∗*^*p* < 0.05.

Vash1 is reported to be expressed preferentially in ECs ([14]; Shibuya et al., 2006). This was true in the WAT, as the expression of vash1 was dominant in CD31-positive cells isolated from the WAT (Figure 5(a)). Moreover, the expression of vash1 in in vitro differentiated adipocytes was comparable to the standard copy number of 1–10^2^ and was negligible (Figure 5(b)). These results highlight the particular role of endothelial vash1 in the expression of insr, irs-1, and irs-2 in the WAT.

### 3.4. Lack of the vash1 Gene Does Not Cause Diabetes Mellitus

**Figure 6:**
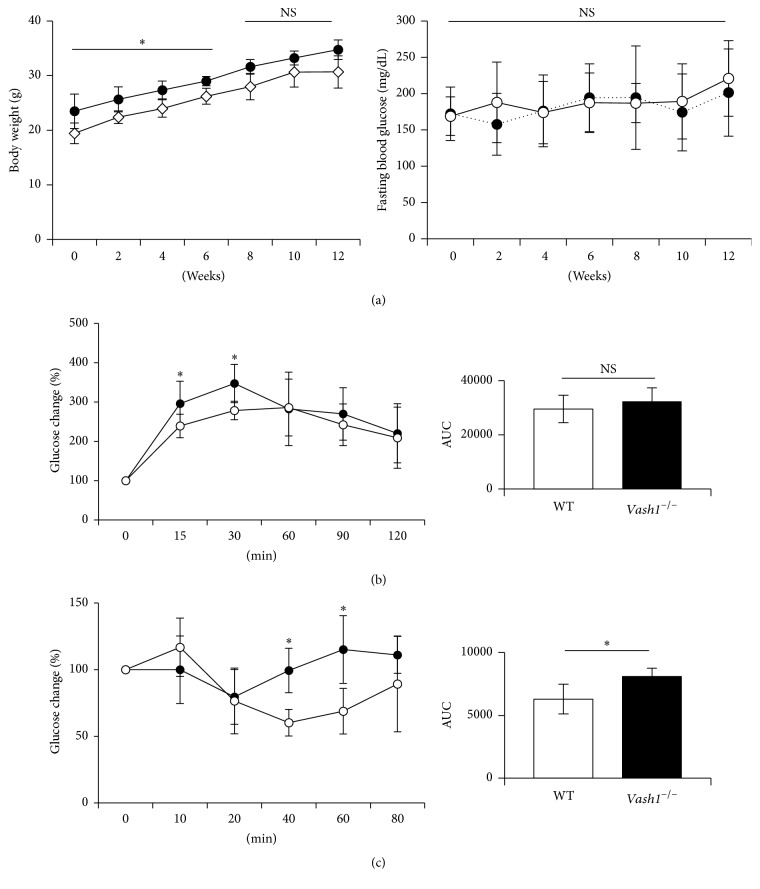
No outbreak of diabetes in* vash1*^−/−^ mice. (a) WT and* vash1*^−/−^ young male mice were fed a HFD, and body weight and fasting blood glucose were compared. Open circle, WT mice; closed circle,* vash1*^−/−^ mice. Means ± SDs are shown (*N* = 7). The statistical significance of differences was calculated by use of the unpaired Student's *t*-test, and a value of *p* < 0.05 was the criterion for significance. (b) After a 12-week HFD feeding, the GTT was performed. Open circle, WT mice; closed circle,* vash1*^−/−^ mice. Area under the curve (AUC) is shown on the right. Means ± SDs are given (*N* = 7). The statistical significance of differences was calculated by performing the unpaired Student's *t*-test, and a value of *p* < 0.05 was the criterion for significance. (c) After a 12-week HFD feeding, the ITT was performed. Open circle, WT mice; closed circle,* vash1*^−/−^ mice. AUC is shown on the right. Means ± SDs are given (*N* = 7). The statistical significance of differences was calculated by use of the unpaired Student's *t*-test, and a value of *p* < 0.05 was taken as the criterion for significance. ^*∗*^*p* < 0.05.

Finally, we tested whether or not the lack of the vash1 gene would be deleterious, causing diabetes. WT mice and* vash1*^−/−^ mice were fed a high-fat diet (HFD) for 12 weeks, with various parameters being monitored.* Vash1*^−/−^ mice were initially heavier than WT mice, but this difference disappeared later. Prior to HFD, we performed GTT and ITT and revealed the tendency of insulin resistance (Supporting Figure  4). We also could not find any differences in the fasting blood glucose level during the HFD feeding period (Figure 6(a)). Plasma adiponectin and leptin concentrations did not show any differences during the HFD feeding period (Supporting Figure  5). After a 12-week HFD feeding, we also performed GTT and ITT. Those examinations revealed that* vash1*^−/−^ mice showed mild glucose intolerance and insulin resistance but never developed overt diabetes (Figures 6(b) and 6(c)).

## 4. Discussion

This is the first demonstration that conventional knockout of the* vash1* gene causes healthy longevity in mice. This finding was unexpected and surprising to us, because we regarded Vash1 as an intrinsic factor that protects vascular ECs from cellular stresses. Indeed, when* vash1* knockout mice are exposed to various stresses, they develop vascular diseases such as diabetic nephropathy and atherosclerotic lesions [16, 17]. Moreover, extensive stresses induced by palaquat administration cause acute lung injury and kill more* vash1* knockout mice than WT mice [15]. Thus, the present unexpected findings forced us to evaluate how the lack of* vash1* gene caused this healthy longevity.

As described earlier, insulin signaling is essential for glucose homeostasis, but it is also involved in longevity. The principal target organs of insulin for glucose homeostasis are the skeletal muscle, the liver, and the adipose tissue. Insulin plays its role in an organ-specific manner: promotion of glucose and fatty acid uptake in the skeletal muscle, suppression of gluconeogenesis in the liver, and inhibition of lipolysis and stimulation of lipid biosynthesis in the adipose tissue [19]. Among these 3 organs, WAT-specific knockout of the* insr* gene results in longevity [9]. Here we revealed that the expression of vash1 dominated in the WAT among these 3 organs and that gene knockout of* vash1* in mice caused significant downregulation of* insr, irs-1, and irs-2* genes in their WAT but not in their liver or skeletal muscle. Foxo1, one of the most widely expressed members of the subfamilies of the family of winged helix forkhead transcription factors, is a downstream substrate of insulin signaling for the determination of lifespan [20], but its expression was not affected in the WAT of the* vash1*^−/−^ mice. The expression of sirt1 and sod2, targets genes of Vash1 in ECs [15], was unchanged in the WAT, probably because their expressions were ubiquitous and not specific to ECs. Taking those findings into account, we propose that vash1 has a distinguishing role in the expression of insr, irs-1, and irs-2 in the WAT and that their expression may be at least one of the causes of longevity of* vash1*^−/−^ mice.

Recent studies on insulin resistance emphasize the presence of inflammation and alteration of certain cytokines in the adipose tissue even when the focus is on the contribution of adipose ECs, and such insulin resistance rather results in a short life [21–25]. Our results on the expression of tnf*α* did not indicate the involvement of inflammation in the* vash1*^−/−^ mice. Alternatively, because of the intimate anatomical arrangement between adipocytes and capillary networks, blood vessels may directly control the adipocyte function. Indeed, alterations in VEGF signaling modify vascular structure/function and change the insulin sensitivity of adipose tissue independent of inflammation [26–29]. But the lifespan and the status of insr, irs-1, and irs-2 expression were not characterized. The present study is the first to indicate the functional interaction between ECs and adipocytes, as vash1 produced by adipose ECs influenced insulin sensitivity by modulating the expression of insr, irs-1, and irs-2 in the WAT. The molecular mechanism explaining how vash1 regulates the expression of insr, irs-1, and irs-2 in the WAT is currently under investigation.

We previously reported that the expression of vash1 decreases with aging [17]. Insulin resistance and the decreased expression of insr, irs-1, and irs-2 in the WAT of* vash1*^−/−^ mice were evident in their early life but disappeared in their late life. These alterations could be explained by this age-associated downregulation of vash1 in the WAT, as the difference in vash1 expression between WT mice and* vash1*^−/−^ mice was large in their early life but diminished in their late life. Then, the question is raised as to whether such changes in the early life of* vash1*^−/−^ mice can influence longevity in their late life. An increasing amount of evidence indicates that nutritional events and/or hormonal alterations during development influence longevity in later life [30]. We therefore think it is possible that insulin resistance due to the decreased expression of insr, irs-1, and irs-2 in WAT in early life might influence the lifespan of* vash1*^−/−^ mice.

As a decrease in vash1 expression makes the vasculature vulnerable to cellular stresses, we were puzzled and wondered why nature would allow a decrease in the expression of such a valuable protein with aging [17]. There must be a reasonable explanation. In accordance with our present observations, we now speculate that vash1 influences not only tolerance toward vascular stresses but also insulin signaling, especially in the WAT, and that one purpose of age-associated downregulation of vash1 might be to afford healthy longevity.

Importantly,* vash1*^−/−^ mice exhibited mild insulin resistance but never developed diabetes even when fed a HFD. This failure was probably because the downregulation of insr, irs-1, and irs-2 in the* vash1*^−/−^ mice was limited to the WAT. As described earlier, there remains a challenging question as to how preserved insulin signaling for glucose homeostasis and reduced insulin signaling for longevity can be balanced in the body. We consider that the prominent function of vash1 in insulin signaling in WAT can be a promising answer to this question.

## 5. Conclusion

The expression of vash1 is involved in normal insulin sensitivity, and the targets of this effect are* insr, irs-1, and irs-2* genes in the WAT. The lack of the* vash1* gene causes mild insulin resistance and that might have contributed to healthy longevity without the development of overt diabetes. Our present findings should open a novel avenue for further research on healthy longevity.

## Supplementary Material

Gross appearance of pancreatic islets in Supporting Figure 1; the basal expression profile of VASH1 in various human organs in Supporting Figure 2; down-regulation of vash1 expression and normalization of insr, irs-1, and irs-2 expressions in WAT with aging in Supporting Figure 3; GTT and ITT prior to HFD in Supporting Figure 4; plasma adiponectin and leptin levels in Supporting Figure 5.

## Figures and Tables

**Table 1:** Biochemical analysis of sera from WT and *vash1*^−/−^ mice.

	WT (*N* = 8)	*Vash1* ^−/−^ (*N* = 6)	*p* value
TP (g/dL)	4.838 ± 0.737	4.600 ± 1.197	0.6795
ALB (g/dL)	2.788 ± 0.557	2.783 ± 0.631	0.9900
BUN (mg/dL)	65.975 ± 47.696	57.850 ± 54.862	0.7779
CRE (mg/dL)	0.288 ± 0.162	0.108 ± 0.037	0.0164
Na (mEq/L)	153.75 ± 3.69	155.67 ± 4.13	0.3896
K (mEq/L)	6.250 ± 1.01	5.883 ± 2.054	0.6998
Cl (mEq/L)	107.63 ± 8.434	114.67 ± 7.005	0.1144
Ca (mg/dL)	9.913 ± 0.954	8.983 ± 0.708	0.0584
IP (mg/dL)	12.475 ± 0.774	11.833 ± 4.514	0.7440
Fe (mg/dL)	84.125 ± 26.712	116.00 ± 33.196	0.0842
AST (IU/L)	137.5 ± 37.4	171.5 ± 93.6	0.4317
ALT (IU/L)	22.875 ± 17.158	40.5 ± 31.15	0.2497
ALP (IU/L)	353.75 ± 86	357.83 ± 56.99	0.9168
LDH (IU/L)	518.5 ± 215.6	561.8 ± 503.4	0.8494
LAP (IU/L)	47.375 ± 7.963	41.333 ± 10.192	0.2590
AMY (IU/L)	3353.13 ± 584.42	4528.33 ± 4351.53	0.5394
CK (IU/L)	435.75 ± 326.84	491.67 ± 319.69	0.7544
ChE (IU/L)	37.625 ± 6.589	31.833 ± 10.722	0.2773
Lip (IU/L)	32.25 ± 27.92	17.33 ± 7.15	0.1844
T-CHOL (mg/dL)	113.875 ± 47.179	65.167 ± 30.656	0.0379
F-CHOL (mg/dL)	29.125 ± 13.840	16.167 ± 7.574	0.0464
E-CHOL (mg/dL)	84.75 ± 33.65	49.00 ± 23.45	0.0373
TG (mg/dL)	55.125 ± 26.867	29.667 ± 18.843	0.0594
PL (mg/dL)	218.25 ± 90.30	121.33 ± 59.51	0.0328
NEFA (mEq/L)	896.00 ± 409.70	937.00 ± 384.02	0.8519
LDL-C (mg/dL)	12.88 ± 7.41	7.00 ± 2.83	0.0689
HDL-C (mg/dL)	53.625 ± 22.507	32.833 ± 20.331	0.0968
T-BIL (mg/dL)	0.053 ± 0.021	0.105 ± 0.034	0.0114
D-BIL (mg/dL)	0.028 ± 0.013	0.057 ± 0.024	0.0311
I-BIL (mg/dL)	0.025 ± 0.020	0.048 ± 0.021	0.0631
TBA (mmol/L)	7.000 ± 7.010	12.667 ± 7.005	0.1626
TL (mg/dL)	325.50 ± 179.09	182.33 ± 89.35	0.2126
GLU (mg/dL)	101.625 ± 48.603	118.000 ± 68.667	0.6310
PA (mg/dL)	1.305 ± 0.351	1.515 ± 0.295	0.2482
LA (mg/dL)	52.063 ± 16.266	44.783 ± 15.497	0.4124
T-KB (mmol/L)	794.88 ± 504.14	937.83 ± 536.65	0.6232

Total protein (TP), albumin (ALB), blood urea nitrogen (BUN), creatinine (CRE), uric acid (UA), sodium (Na), potassium (K), chloride (Cl), calcium (Ca), inorganic phosphorous (IP), iron (Fe), aspartate aminotransferase (AST), alanine aminotransferase (ALT), alkaline phosphatase (ALP), lactate dehydrogenase (LDH), leucine aminopeptidase (LAP), amylase (AMY), creatine kinase (CK), choline esterase (ChE), lipase (Lip), total cholesterol (T-CHOL), free cholesterol (F-CHOL), esterified cholesterol (E-CHOL), triglyceride (TG), phospholipid (PL), nonesterified fatty acid (NEFA), low-density lipoprotein cholesterol (LDL-C), high-density lipoprotein cholesterol (HDL-C), total bilirubin (T-BIL), direct bilirubin (D-BIL), indirect bilirubin (I-BIL), total bile acid (TBA), total lipid (TL), blood glucose (GLU), pyruvic acid (PA), lactic acid (LA), and total ketone body (T-KB). Means ± SDs and *p* values are shown.

**Table 2:** Primer list.

Genes	Forward (5′ → 3′)	Reverse (5′ → 3′)
Vash1	GATTCCCATACCAAGTGTGCC	ATGTGGCGGAAGTAGTTCCC
Insr	ATGAGGCCAACCTTCCTGGAA	ACGGGACATTCTCCATGTCT
Irs-1	AGCCCAAAAGCCCAGGAGAATA	TTCCGAGCCAGTCTCTTCTCTA
Irs-2	AGTAAACGGAGGTGGCTACA	AAGCTGCTGAGAAGTCAGGT
Sod2	GGTCGCTTACAGATTGCT	CTCCCAGTTGATTACATTCC
Sirt1	AGTTCCAGCCGTCTCTGTGT	CTCCACGAACAGCTTCACAA
FoxO1	GCTGGGTGTCAGGCTAAGAG	TGGACTGCTCCTCAGTTCCT
Tnf*α*	GGGACAGTGACCTGGACTGT	AGGCTGTGCATTGCACCTCA
*β*-Actin	TCGTGCGTGACATCAAAGAG	TGGACAGTGAGGCCAGGATG
