# Supplementary material for: Knockout of Vasohibin-1 Gene in Mice Results in Healthy Longevity with Reduced Expression of Insulin Receptor, Insulin Receptor Substrate 1, and Insulin Receptor Substrate 2 in Their White Adipose Tissue
Source: J Aging Res. 2017 Mar 6;2017:9851380. doi: 10.1155/2017/9851380 (PMC5358453; doi:10.1155/2017/9851380)

**Supporting Information**

Supporting Figure 1: Gross appearance of pancreatic islets.

Pancreas tissues were obtained and Hematoxylin-Eosin staining was performed. Bars: 100 μm.

Supporting Figure 2: The basal expression profile of VASH1 in various human organs. The basal expression profile of VASH1 in various human organs was obtained from Genotype-Tissue Expression Portal (http://www.broadinstitute.org/gtex/)

Supporting Figure 3: Down-regulation of vash1 expression and normalization of insr, irs-1, and irs-2 expressions in WAT with aging

A: Total RNA was isolated from the WT and *vash1^-/-^* WAT and liver of young male mice, and the expression of vash1 was compared. B: Total RNA was isolated from the WT and *vash1^-/-^* WAT of old male mice, and the expression of insr, irs-1, and irs-2 was compared between WT and *vash1^-/^* mice. In “A” and “B,” the means±SDs are shown, the statistical significance of differences was calculated by use of the unpaired Student’s t test, and a value of p < 0.05 was the criterion for significance.

Supporting Figure 4: GTT and ITT prior to HFD

Prior to HFD, GTT and ITT were performed. Open circle, WT mice; closed circle, *vash1^-/-^* mice. Means±SDs are given (N=7). The statistical significance of differences was calculated by performing the unpaired Student’s t test, and a value of p < 0.05 was the criterion for significance. Area under the curve (AUC) is shown on the right. There were no statistical significant differences in AUCs.

Supporting Figure 5: Plasma adiponectin and leptin levels.

During the HFD period, plasma adiponectin and leptin levels were determined. Open circle, WT mice; closed circle, *vash1^-/-^* mice (N=7). There were no statistical significant differences.


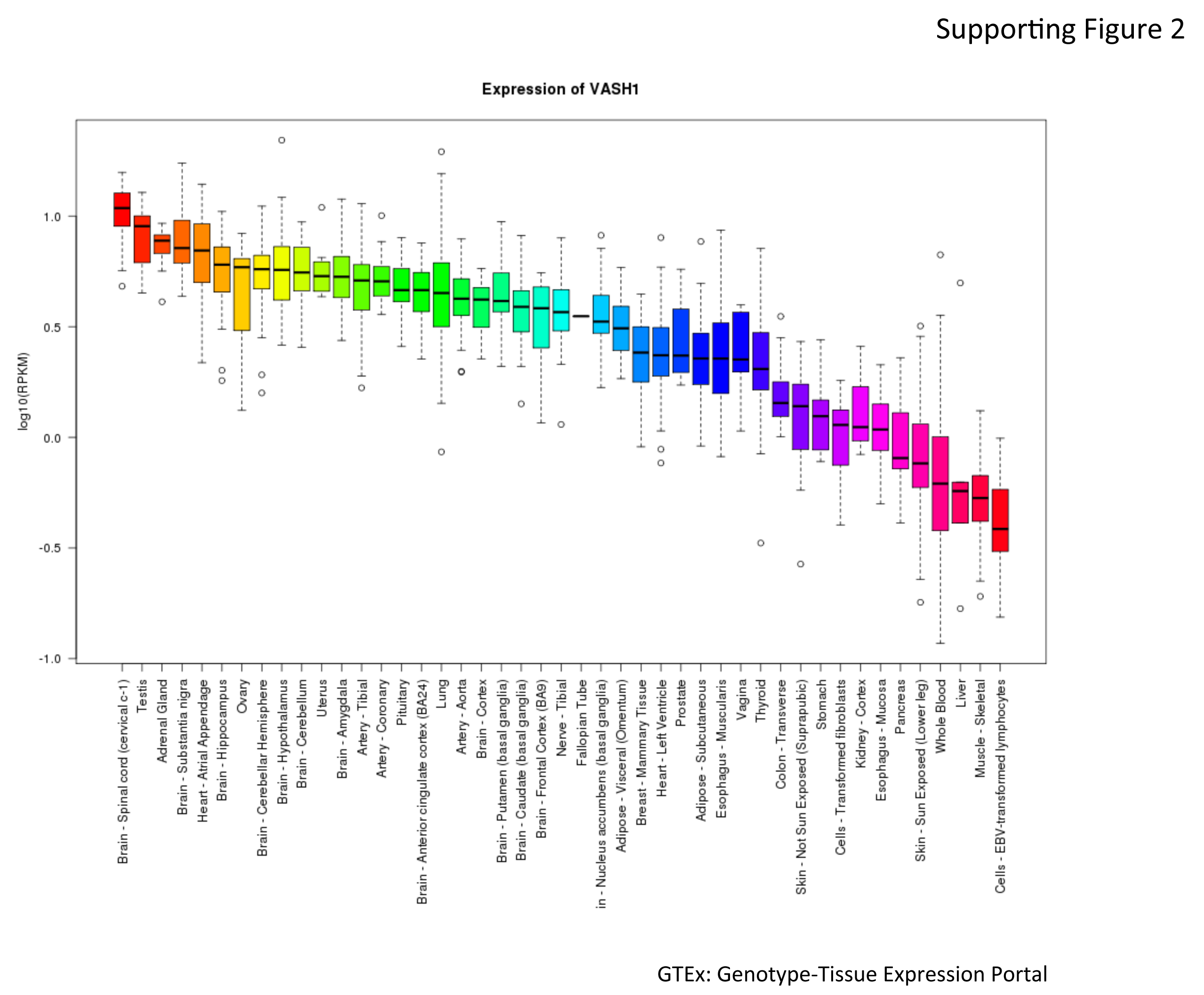


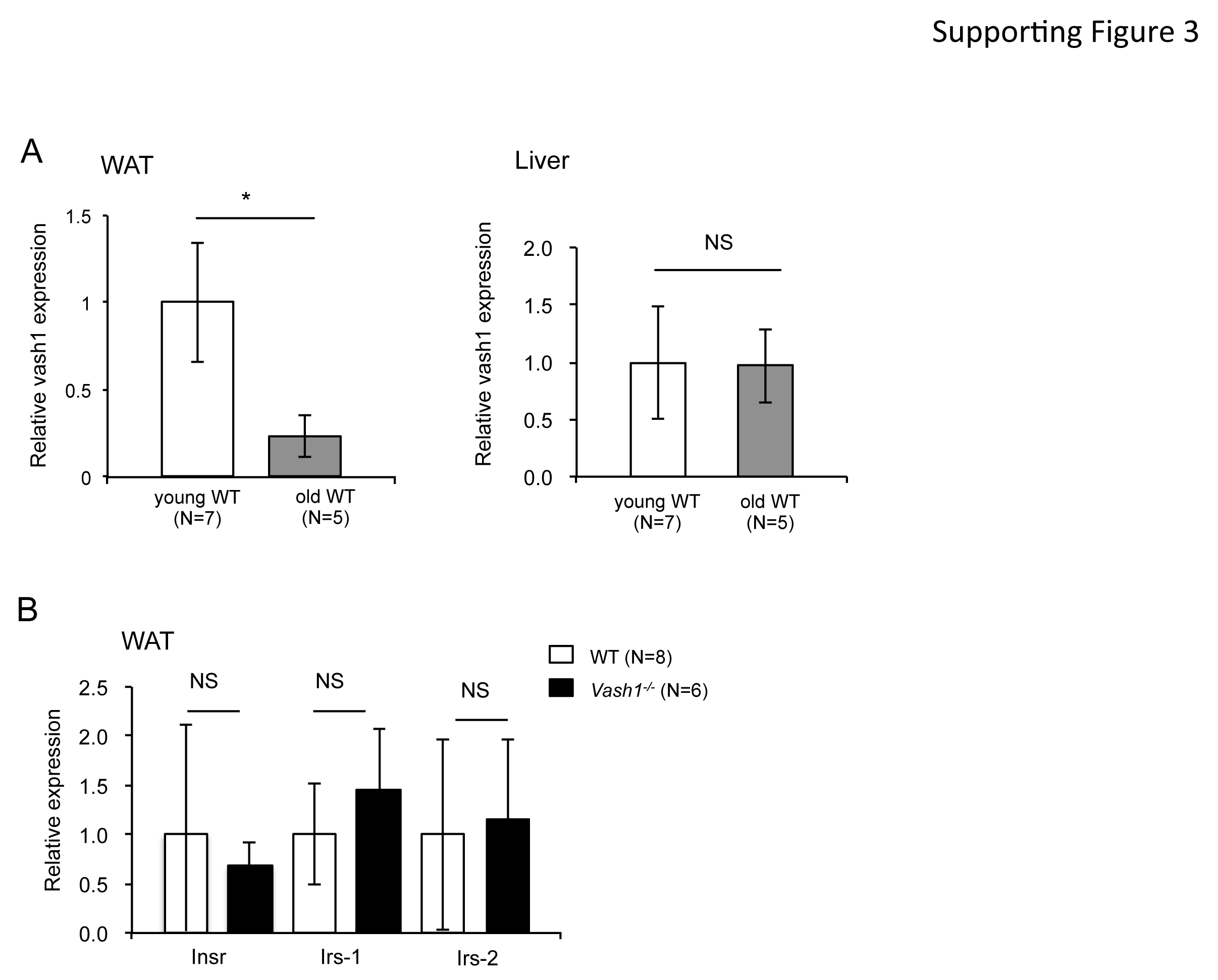


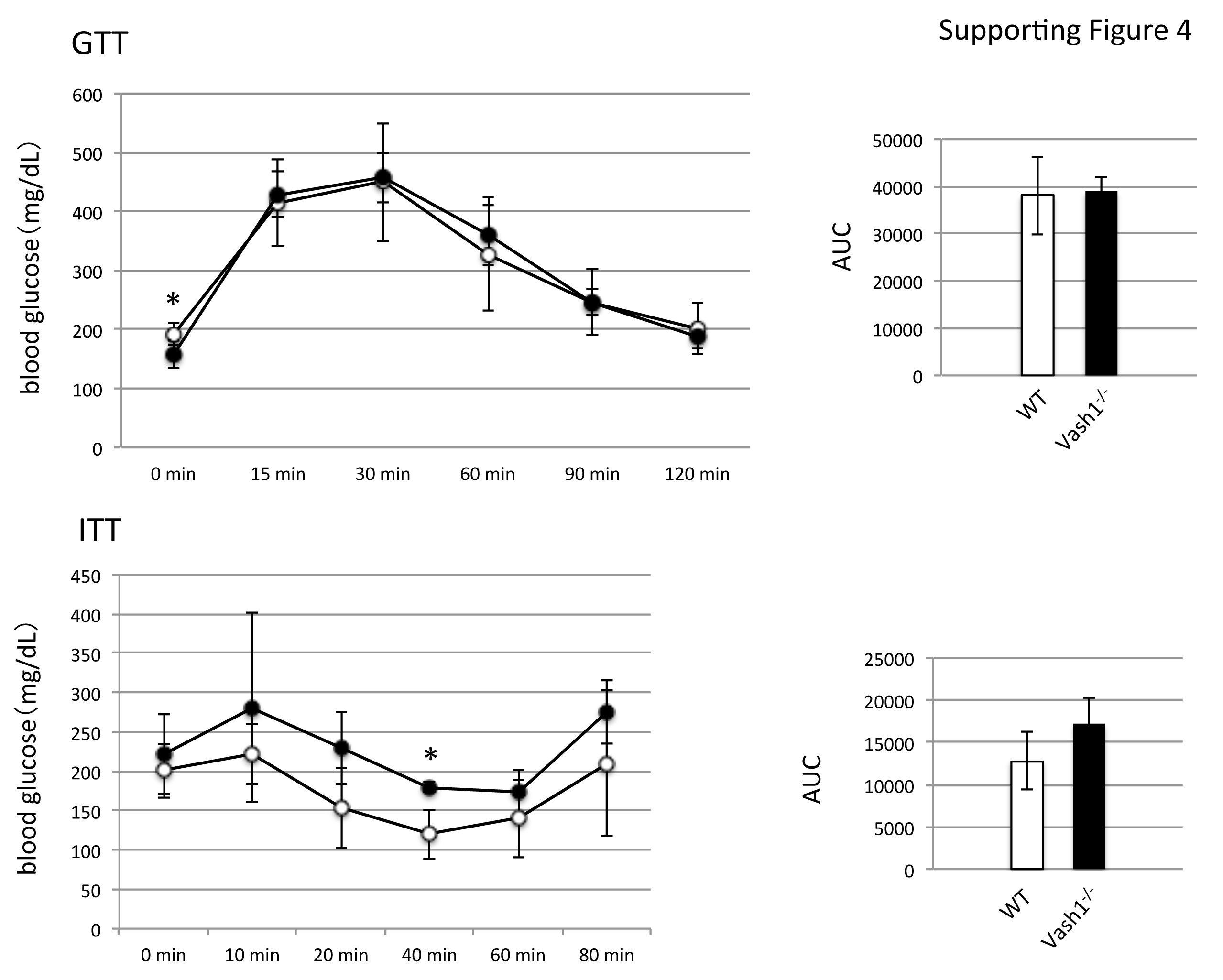


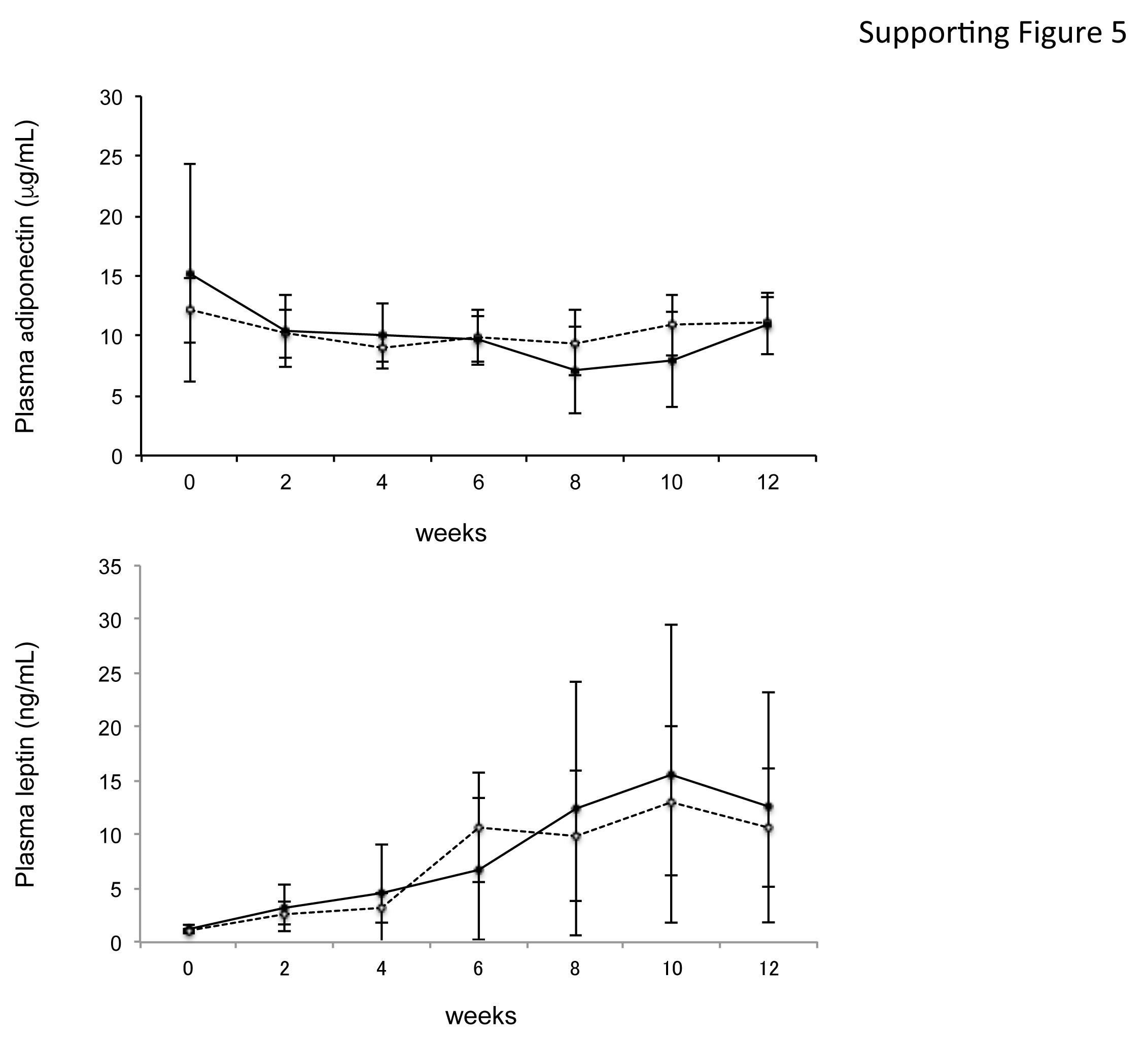

Supplement: Supplementary file 1 — Gross appearance of pancreatic islets in Supporting Figure 1; the basal expression profile of VASH1 in various human organs in Supporting Figure 2; down-regulation of vash1 expression and normalization of insr, irs-1, and irs-2 expressions in WAT with aging in Supporting Figure 3; GTT and ITT prior to HFD in Supporting Figure 4; plasma adiponectin and leptin levels in Supporting Figure 5. [file 9851380.f1.docx]
